# Comparison of Cardiometabolic Profiles in Patients with Nonfunctioning Adrenal Incidentalomas and Mild Autonomous Cortisol Secretion

**DOI:** 10.3390/jcm15166378

**Published:** 2026-08-18

**Authors:** Rabia Eker Akilli, Ulcaz Perihan Aksoydan, Omer Tepe, Gamze Akkus, Onur Sinan Deveci, Anil Akray, Imam Gunay

**Affiliations:** 1 Department of Cardiology, Faculty of Medicine, Cukurova University, Adana 01330, Turkey; onurdeveci@yahoo.co.uk; 2 Department of Endocrinology, Faculty of Medicine, Mersin University, Mersin 33343, Turkey; ulcazaksoydan@gmail.com; 3 Osmaniye Education and Research Hospital, Osmaniye 80010, Turkey; omer.tepe0133@gmail.com; 4 Department of Endocrinology, Faculty of Medicine, Cukurova University, Adana 01330, Turkey; tugrulgamze@hotmail.com; 5 Iskenderun State Hospital, Hatay 31230, Turkey; anl_akry@hotmail.com; 6 Yozgat City Hospital, Yozgat 66100, Turkey; dr.imamgunay@hotmail.com

**Keywords:** mild autonomous cortisol secretion, adrenal incidentaloma, echocardiography, global longitudinal strain, cardiometabolic risk, subclinical hypercortisolism

## Abstract

**Objective**: To compare the cardiometabolic characteristics of patients with mild autonomous cortisol secretion (MACS) and nonfunctioning adrenal incidentalomas (NFAI), with particular emphasis on their biochemical and echocardiographic profiles. **Methods**: This retrospective study included 67 patients with adrenal incidentalomas, classified as NFAI (*n* = 32) or MACS (*n* = 35) according to the post-1-mg dexamethasone suppression test (DST) cortisol concentration. Clinical characteristics, hormonal profiles, metabolic parameters, conventional echocardiographic measurements, and global longitudinal strain (GLS) were compared between the groups. Associations between GLS and biochemical parameters were evaluated using correlation analyses, with Benjamini–Hochberg false discovery rate correction applied for multiple testing. **Results**: Compared with the NFAI group, patients with MACS had lower adrenocorticotropic hormone (ACTH) (13.0 ± 7.1 vs. 18.6 ± 9.7 pg/mL, *p* = 0.008) and dehydroepiandrosterone sulfate (DHEA-S) concentrations (46.4 ± 31.6 vs. 82.8 ± 48.0 µg/dL, *p* < 0.001), as well as higher post-1-mg DST cortisol concentrations (3.4 ± 1.3 vs. 1.2 ± 0.5 µg/dL, *p* < 0.001). Triglyceride concentrations were also higher in the MACS group (165.3 ± 70 vs. 129.4 ± 90.2 m g/dL, *p* = 0.004). Most conventional echocardiographic parameters were comparable between the groups. GLS did not differ significantly between NFAI and MACS patients (21.92 ± 2.2 vs. 21.94 ± 1.9, respectively; mean difference: 0.0, 95% CI: −1.0 to 1.0, *p* = 0.846). Nominal correlations between GLS and selected biochemical parameters did not remain statistically significant after false discovery rate correction. **Conclusions**: MACS was associated with distinct endocrine and metabolic characteristics compared with NFAI; however, no significant between-group difference in GLS was observed. These cross-sectional findings do not establish myocardial dysfunction or a temporal relationship between cortisol autonomy and cardiovascular alterations. Larger prospective longitudinal studies are required to clarify the cardiovascular implications of MACS.

## 1. Introduction

Adrenal incidentalomas are adrenal masses detected unexpectedly during imaging performed for indications unrelated to suspected adrenal disease, and their identification has increased with the widespread use of cross-sectional imaging [1,2,3]. According to the 2023 European Society of Endocrinology guideline, patients without overt features of Cushing syndrome who have a serum cortisol concentration > 1.8 µg/dL (>50 nmol/L) after the 1 mg overnight dexamethasone suppression test (DST) are classified as having mild autonomous cortisol secretion (MACS), following confirmation of ACTH-independent cortisol secretion. In contrast, nonfunctioning adrenal incidentaloma (NFAI) is characterized by the absence of clinically relevant hormone excess and adequate cortisol suppression after the 1 mg DST (≤1.8 µg/dL [≤50 nmol/L]) [1,2].

The reported prevalence of MACS varies because of differences in study populations, diagnostic thresholds, assay methods, and evolving terminology [1,2,4]. Cortisol autonomy is increasingly considered a biological continuum rather than a strictly dichotomous condition. Observational studies have reported higher frequencies of hypertension, impaired glucose metabolism, dyslipidemia, and obesity among patients with MACS than among those with NFAI [4,5,6]. Associations with cardiovascular events and mortality have also been described, although the magnitude of these associations varies across cohorts and may be influenced by age, sex, the degree of cortisol autonomy, diagnostic definitions, and conventional cardiovascular risk factors [7,8,9,10].

Prolonged cortisol exposure may contribute to cardiometabolic abnormalities through alterations in vascular tone, sodium homeostasis, insulin sensitivity, lipid metabolism, endothelial function, and glucocorticoid- and mineralocorticoid-receptor signaling [11,12,13,14]. Increased left ventricular mass, impaired diastolic function, arterial stiffness, and other markers of cardiovascular remodeling have been reported in some adrenal incidentaloma cohorts [5,15,16,17]. However, these findings have not been consistent, and evidence regarding myocardial deformation assessed using global longitudinal strain (GLS) remains limited. Whether MACS is associated with alterations in GLS independently of accompanying demographic, metabolic, and clinical factors therefore remains uncertain.

This study aimed to compare the clinical, metabolic, hormonal, and echocardiographic characteristics of patients with MACS and NFAI, with particular emphasis on GLS and conventional echocardiographic parameters, and to explore the relationships between GLS and selected biochemical variables.

## 2. Methods

### 2.1. Study Design and Population

This cross-sectional observational study initially screened 94 patients evaluated for adrenal incidentalomas in the endocrinology and cardiology outpatient clinics. Of these, 27 were excluded because of overt Cushing syndrome (*n* = 7), pheochromocytoma (*n* = 5), primary aldosteronism (*n* = 3), obstructive coronary artery disease (*n* = 7), or significant valvular heart disease (*n* = 5). The cardiovascular exclusion criteria included clinically significant cardiovascular disease, defined as obstructive coronary artery disease, previous myocardial infarction or acute coronary syndrome, previous coronary revascularization, inducible myocardial ischemia, regional wall motion abnormalities, heart failure, cardiomyopathy, or significant valvular heart disease. In 12 patients of the study cohort had incidentally detected non-obstructive coronary atherosclerosis, defined as coronary plaques causing less than 50% stenosis on coronary computed tomography angiography. None of these patients had a history of myocardial infarction or acute coronary syndrome, previous revascularization, inducible ischemia, regional wall motion abnormalities, or an indication for revascularization. After excluding these 12 patients, the sensitivity analysis findings remained significantly consistent with the primary analysis findings in terms of both statistical significance and effect size, so these patients were not excluded and were included in the final analysis cohort. No patients were excluded from the study due to potential medication use or conditions that could affect DST, and therefore no changes were made to the final study sample. Ultimately, 67 eligible patients were included in the final analysis. Medication histories were reviewed for the use of antihypertensive, glucose-lowering, lipid-lowering, and antiplatelet therapies. Because medication categories were not mutually exclusive, individual patients could be included in more than one treatment category.

All patients underwent clinical evaluation for features of overt Cushing syndrome and standard hormonal testing to assess adrenal function and exclude other causes of hormone excess. For the 1 mg overnight DST, dexamethasone was administered at 23:00, and serum cortisol was measured the following morning between 08:00 and 09:00. Before testing, medication histories and clinical conditions potentially affecting dexamethasone metabolism, cortisol-binding globulin concentrations, or cortisol measurement were reviewed. Relevant medications included exogenous glucocorticoids administered by any route; oral estrogen-containing preparations; antiepileptic drugs such as carbamazepine, phenytoin, phenobarbital, and primidone; rifampicin or rifapentine; and strong CYP3A4 inhibitors, including azole antifungals, ritonavir, clarithromycin, and diltiazem. None of the included patients were receiving these medications at the time of testing.

**Figure 1:**
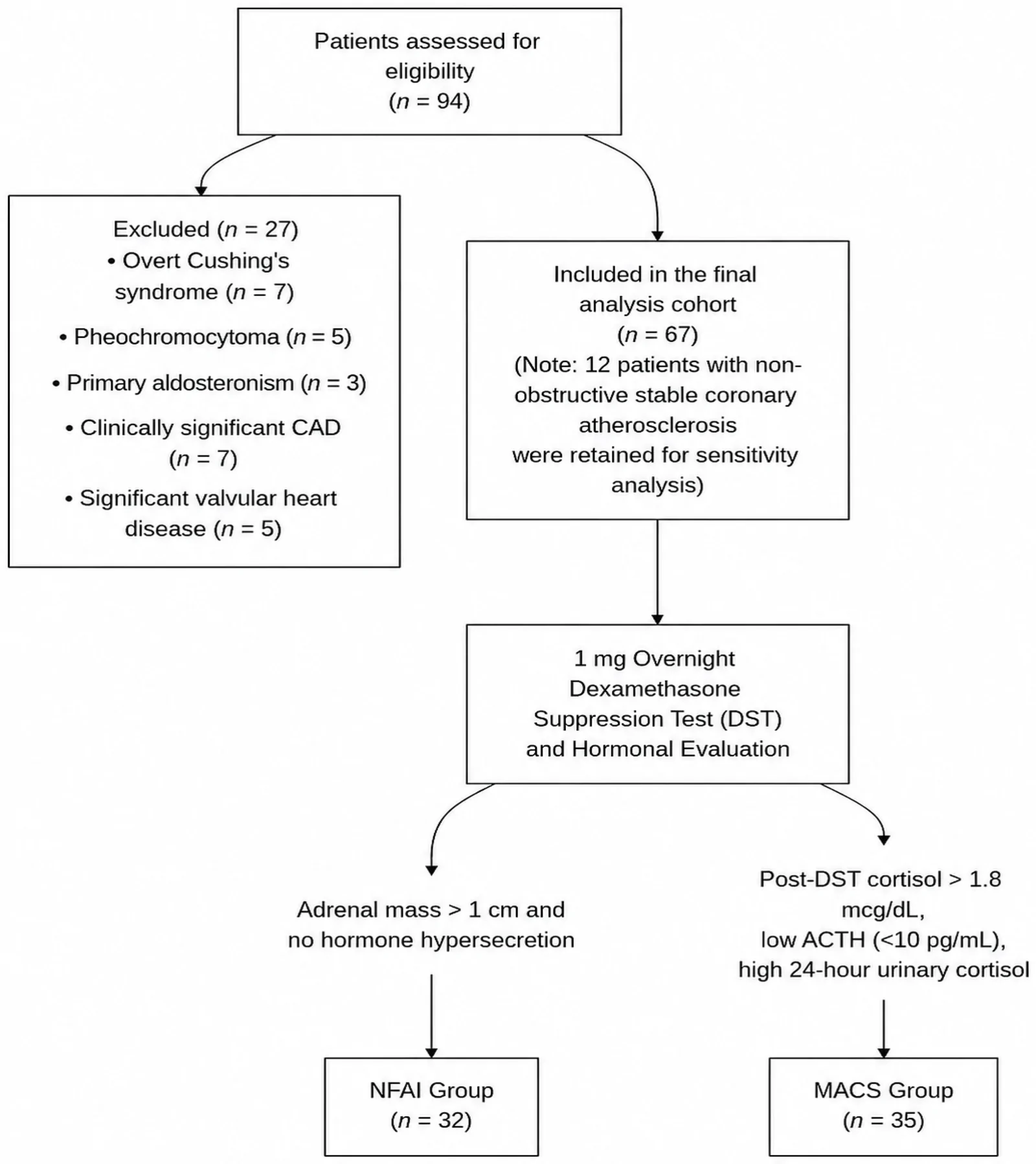
Flowchart of the study. CAD: Coronary artery disease, NFAI: Nonfunctioning adrenal incidentaloma, MACS: Mild autonomous cortisol secretion.

In accordance with the 2023 European Society of Endocrinology/European Network for the Study of Adrenal Tumors guideline [1], patients with an adrenal mass > 1 cm, no evidence of clinically relevant hormone excess, and a post-1-mg DST cortisol concentration ≤ 1.8 µg/dL (≤50 nmol/L) were classified as having NFAI (*n* = 32). Patients without clinical features of overt Cushing’s syndrome who had a post-1-mg DST serum cortisol concentration > 1.8 µg/dL (>50 nmol/L) were classified as having MACS (*n* = 35). Morning plasma ACTH concentrations, available for all participants, were measured using a two-step sandwich chemiluminescent immunoassay on the MAGLUMI X3 Fully Automated Chemiluminescence Immunoassay Analyzer. Because the guideline does not define a universal numerical ACTH cutoff and reference intervals are assay dependent, ACTH was interpreted as a continuous biochemical variable within the appropriate clinical context and used as supportive evidence of ACTH-independent cortisol secretion. An ACTH concentration below the assay-specific reference interval was not required as a separate mandatory classification criterion; group allocation was based primarily on the post-1-mg DST cortisol concentration. Additional assessments, including repeat 1 mg DST, were performed when clinically indicated, and their results were considered supportive rather than mandatory diagnostic criteria. Owing to the retrospective study design, the results of these tests were not uniformly available and were therefore not included in the analyses. The participant-selection and diagnostic-classification process (flowchart of the study) is presented in Figure 1.

### 2.2. Biochemical and Hormonal Assessment

Venous blood samples were collected in the morning (08:00–09:00) after an overnight fast. The following parameters were measured: fasting glucose, glycated hemoglobin (HbA1c), insulin, lipid profile [total cholesterol, LDL, triglycerides (TG)], serum creatinine, alanine aminotransferase (ALT), sodium, potassium, and thyroid-stimulating hormone (TSH). Serum cortisol was measured using a paramagnetic particle chemiluminescent immunoassay on the UniCel DxI 800 Access Immunoassay System (Beckman Coulter Inc., Brea, CA, USA). Other hormonal analyses included aldosterone, renin activity, and DHEA-S.

In addition, 24 h urinary concentrations of free cortisol, metanephrine, and normetanephrine were measured using standard immunoassay or LC–MS/MS methods. Urinary metanephrine and normetanephrine measurements were performed as part of the routine evaluation for pheochromocytoma. Because patients with pheochromocytoma were excluded and the values in the eligible cohort remained within the reference range, these parameters were not included in the statistical analyses. Insulin resistance was calculated using the homeostasis model assessment of insulin resistance (HOMA-IR). Estimated glomerular filtration rate (eGFR) was calculated with the CKD-EPI formula.

### 2.3. Imaging and Echocardiographic Evaluation

Adrenal lesions were evaluated primarily using computed tomography (CT) to determine lesion size and imaging characteristics. The evaluation of adrenal lesions was primarily performed using computed tomography (CT), while magnetic resonance imaging (MRI) was utilized in selected cases based on institutional availability, patient-specific clinical factors, or contraindications (such as contrast allergy or renal dysfunction).

Comprehensive transthoracic echocardiographic examinations were performed using a commercially available echocardiography system (Vivid E95 cardiovascular ultrasound system GE Vingmed Ultrasound AS, Horten, Norway) equipped with a phased-array transducer. Images were acquired with patients in the left lateral decubitus position and sinus rhythm, in accordance with current echocardiographic recommendations [18,19]. Standard two-dimensional, M-mode, pulsed-wave Doppler, and tissue-Doppler images were obtained. Left ventricular ejection fraction was calculated using the biplane Simpson method. Left ventricular mass was calculated using linear measurements and indexed to body surface area. Relative wall thickness was calculated as twice the posterior wall thickness divided by the left ventricular end-diastolic diameter. Left atrial volume was measured using the biplane area–length method and indexed to body surface area.

Mitral inflow velocities were measured using pulsed-wave Doppler at the tips of the mitral leaflets, including peak early- (E) and late- (A) diastolic velocities and the E/A ratio. Tissue-Doppler imaging was performed at the septal and lateral mitral annuli to measure early-diastolic (e′), late-diastolic (a′), and systolic (s′) velocities. The average E/e′ ratio was calculated using the mitrel E and lateral e′ velocities.

Two-dimensional speckle-tracking analysis was performed offline using EchoPAC software, version 202, (GE Vingmed Ultrasound AS, Horten, Norway). Apical four-chamber, two-chamber, and long-axis views were acquired at a frame rate of 40–90 frames/s. Endocardial borders were initially identified automatically and manually adjusted when necessary to ensure accurate tracking throughout the cardiac cycle. Tracking quality was visually assessed for each myocardial segment. Segments with inadequate tracking were manually readjusted; segments that remained inadequately tracked after correction were rejected. Examinations with fewer than 17 analyzable segments were excluded from GLS analysis. Left ventricular GLS was calculated as the average peak systolic longitudinal strain across the analyzable segments from the three apical views. GLS was reported as a signed percentage, with more negative values indicating better systolic deformation and less negative values indicating impaired systolic deformation. All echocardiographic and strain analyses were performed by a single experienced cardiologist who was blinded to the patients’ clinical and laboratory data. Formal intraobserver and interobserver reproducibility analyses were not performed.

### 2.4. Statistical Analysis

Statistical analyses were performed using IBM SPSS Statistics version 20.0 (IBM Corp., Armonk, NY, USA). The distribution of continuous variables was assessed using the Kolmogorov–Smirnov test. Continuous variables were expressed as mean ± standard deviation or median [interquartile range], as appropriate, and categorical variables as number (percentage).

Between-group comparisons were performed using the independent-samples *t*-test or Mann–Whitney *U* test for continuous variables and the chi-square test or Fisher’s exact test for categorical variables, as appropriate. Associations between GLS and selected clinical, biochemical, hormonal, and echocardiographic variables were evaluated using Pearson or Spearman correlation coefficients according to data distribution. To account for multiple testing across the correlation analyses, *p*-values were adjusted using the Benjamini–Hochberg false discovery rate procedure.

A multivariable linear regression model was constructed with GLS as the dependent variable. Age, sex, body mass index, hypertension, diabetes mellitus, diagnostic group (MACS versus NFAI), and post-1-mg DST cortisol concentration as a continuous variable were included as prespecified clinically relevant covariates. Regression results were reported as regression coefficients with 95% confidence intervals and *p*-values. Multicollinearity was assessed using variance inflation factors. Residual distribution, homoscedasticity, and multicollinearity were assessed to evaluate model assumptions, with multicollinearity examined using variance inflation factors.

To assess the potential influence of coronary atherosclerosis on GLS and the principal between-group comparisons, a sensitivity analysis was performed after excluding the 12 patients with non-obstructive coronary atherosclerosis. Because the results were materially consistent with those of the primary analysis, these patients were retained in the final analysis cohort. All tests were two-tailed. A *p*-value < 0.05 was considered statistically significant.

### 2.5. Ethical Considerations

The study protocol was approved by the institutional ethics committee with decision number 117/9. Written informed consent was obtained from all participants prior to inclusion, and all procedures were conducted in accordance with the Declaration of Helsinki.

## 3. Results

The study population comprised 67 patients with adrenal incidentalomas, including 35 patients with MACS (52.2%) and 32 with NFAI (47.8%). The mean age was 58.0 ± 8.4 years, and 44 patients (65.7%) were female. According to BMI categories, 13 patients (19.4%) had normal weight, 27 (40.3%) were overweight, and 27 (40.3%) were obese. Overall, 52 patients (77.6%) had at least one comorbidity. The clinical characteristics of the study population are presented in Table 1.

Among the 35 patients with hypertension, 28 (80.0%) were receiving antihypertensive treatment. Of the 22 patients with diabetes mellitus, 14 (63.6%) were receiving glucose-lowering therapy, and 3 of the 8 patients with hyperlipidemia (37.5%) were receiving lipid-lowering therapy. Among the 12 patients with non-obstructive coronary atherosclerosis, seven (58.3%) were receiving low-dose acetylsalicylic acid as antiplatelet therapy. Treatment categories were not mutually exclusive; therefore, individual patients could be represented in more than one category.

A sensitivity analysis excluding the 12 patients with non-obstructive coronary atherosclerosis yielded results materially consistent with those of the primary analysis. In particular, the absence of a statistically significant difference in GLS between the MACS and NFAI groups remained unchanged. Therefore, these patients were retained in the final analysis cohort.

The biochemical characteristics of the study population are summarized in Table 2. CT was used to evaluate adrenal lesions in 55 patients (82.1%), whereas MRI was used in 12 (17.9%). The mean adrenal lesion size was 26.6 ± 10.2 mm.

Comparisons of clinical, biochemical, hormonal, imaging, and GLS parameters between the NFAI and MACS groups are presented in Table 3. Triglyceride concentrations were higher in the MACS group than in the NFAI group (median, 154 [106–222] vs. 102 [78.5–148.5] mg/dL; *p* = 0.004). As expected from the diagnostic classification, post-1-mg DST cortisol concentrations were higher in the MACS group (3.4 ± 1.3 vs. 1.2 ± 0.5 µg/dL; *p* < 0.001), whereas ACTH (13.0 ± 7.1 vs. 18.6 ± 9.7 pg/mL; *p* = 0.008) and DHEA-S concentrations (46.4 ± 31.6 vs. 82.8 ± 48.0 µg/dL; *p* < 0.001) were lower. Adrenal lesion size was also greater in the MACS group (29.4 ± 11.4 vs. 23.6 ± 7.9 mm; *p* = 0.019). No statistically significant between-group difference was detected in GLS (−21.9 ± 1.9% in the MACS group vs. −21.9 ± 2.2% in the NFAI group; mean difference, 0.0, 95% CI, −1.0 to 1.0%; *p* = 0.846).

Conventional echocardiographic parameters are compared in Table 4. Most measurements, including left atrial dimensions and volume, left ventricular internal diameters, LVEF, left ventricular mass index, relative wall thickness, E/A ratio, E/e′ ratio, and tissue-Doppler velocities, did not differ significantly between the groups (all *p* > 0.05). Mitral E-wave velocity was lower in the MACS group than in the NFAI group (56.9 ± 9.2 vs. 63.3 ± 12.3 cm/s; *p* = 0.019). Interventricular septal thickness also differed between the groups (median, 10 [9–11] vs. 10 [9–10] mm; *p* = 0.040), whereas posterior wall thickness and the remaining structural parameters were comparable.

**Figure 2:**
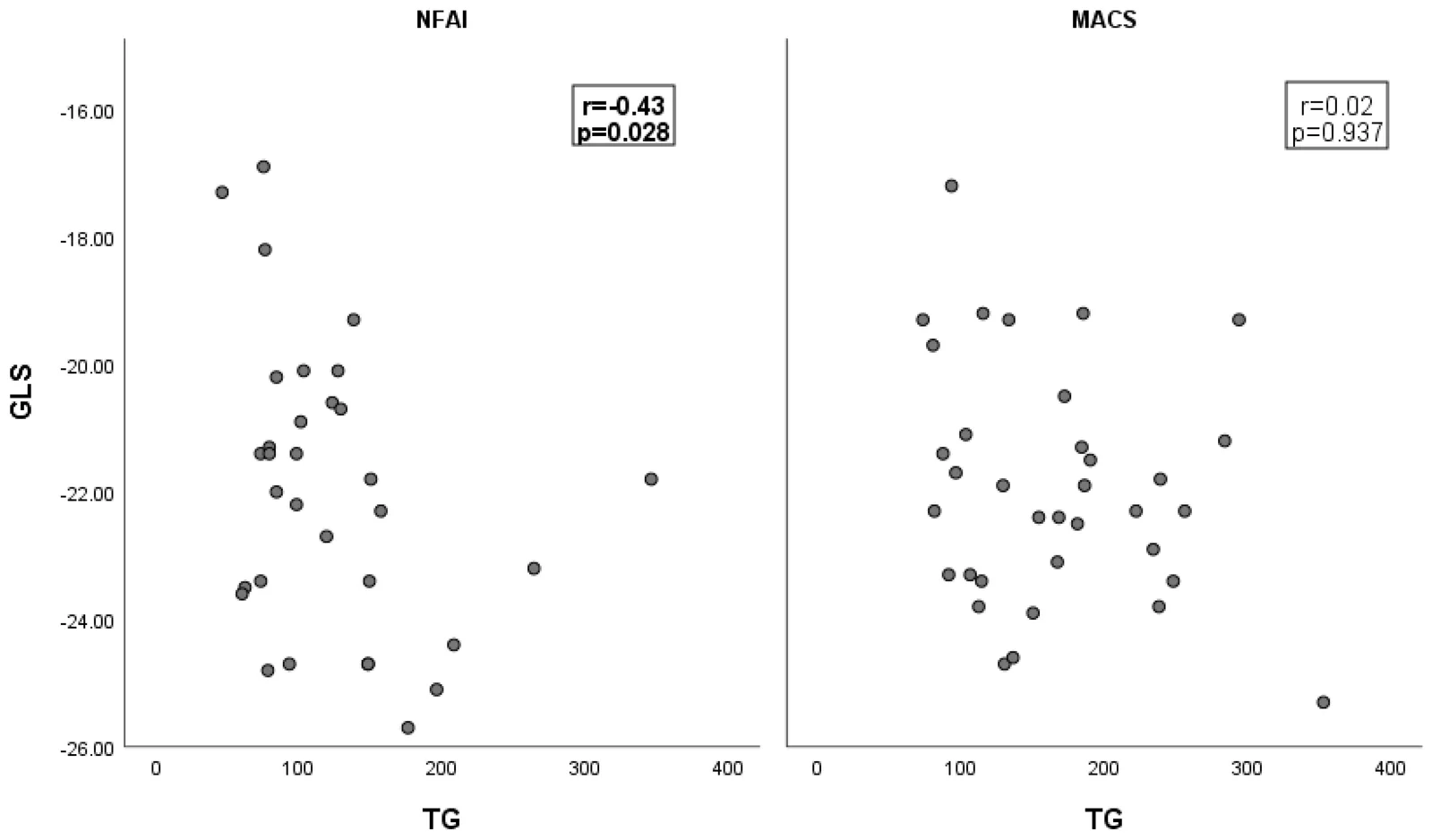
Relationship between TG levels and GLS in the study groups.

In unadjusted analyses of the entire cohort, nominal correlations were observed between GLS and estimated glomerular filtration rate (r = 0.36, *p* = 0.003), glucose (r = −0.25, *p* = 0.040), HbA1c (r = −0.25, *p* = 0.042), and LDL cholesterol (r = 0.25, *p* = 0.046) (Table 5). In the NFAI subgroup, triglyceride concentration showed a nominal negative correlation with GLS (r = −0.43, *p* = 0.028; Figure 2). However, none of these correlations remained statistically significant after Benjamini–Hochberg false discovery rate correction. Accordingly, these unadjusted findings were considered exploratory.

Figure 2 illustrates the subgroup-specific relationships between triglyceride levels and GLS measurements; the nominal association observed in the NFAI group did not remain significant after FDR correction.

To account for potential confounders, a multivariable linear regression analysis was performed with GLS as the dependent variable, adjusting for age, sex, BMI, hypertension, diabetes mellitus, group status (MACS vs. NFAI), and continuous post-1-mg DST cortisol levels. After adjusting for these covariates, left ventricular GLS did not differ significantly between the NFAI and MACS groups (*β* = 0.110, *p* = 0.463). Among all covariates, age (*p* < 0.001) and diabetes mellitus (*p* = 0.006) emerged as significant independent predictors of GLS. Multicollinearity diagnostics revealed no collinearity issues, with all VIF values remaining below 2.5 (Table 6).

## 4. Discussion

In the present study, hormonal and metabolic characteristics, conventional echocardiographic measures, and myocardial deformation parameters were compared between patients with MACS and those with NFAI. Patients with MACS had higher post-1-mg DST cortisol and triglyceride concentrations, lower ACTH and DHEA-S concentrations, and larger adrenal lesions. However, left ventricular GLS did not differ significantly between the groups, and most conventional structural, systolic, and diastolic echocardiographic measures were also similar. Therefore, although MACS was characterized by a distinct endocrine and metabolic profile, this profile was not accompanied by a detectable difference in myocardial deformation in this cohort.

The definition and clinical interpretation of MACS have evolved substantially. According to the 2023 European Society of Endocrinology guideline, MACS is defined by a serum cortisol concentration > 1.8 μg/dL (>50 nmol/L) after the 1 mg DST in patients without overt clinical features of Cushing syndrome, together with confirmation of ACTH-independent cortisol secretion [1]. Post-1-mg DST cortisol is also viewed as a continuous indicator of cortisol autonomy. Accordingly, clinical management is based not solely on biochemical classification but also on potentially cortisol-related comorbidities, age, general health, and patient preferences [1,2]. Variations in diagnostic thresholds, biochemical assessment methods, patient selection, and comorbidity profiles may partly explain the heterogeneity in the reported prevalence and cardiovascular consequences of MACS [5,16].

The higher triglyceride concentrations observed in the MACS group are consistent with previous reports describing an unfavorable cardiometabolic phenotype in autonomous cortisol secretion [20,21]. Nevertheless, recent evidence indicates considerable phenotypic heterogeneity. Chen et al. reported differences in cardiovascular risk markers among patients with adrenal incidentalomas and MACS but showed that risk could not be defined by any single biochemical or clinical measure [5]. Similarly, comprehensive endocrine and metabolic phenotyping by the ENSAT EURINE-ACT investigators revealed complex relationships among cortisol autonomy, steroid metabolism, and established cardiometabolic risk factors [22]. Collectively, these observations suggest that the clinical expression of MACS may reflect an interaction between cortisol exposure and individual susceptibility. Therefore, our triglyceride findings should be interpreted cautiously. Although altered cortisol secretion may contribute to insulin resistance and lipid dysregulation, triglyceride concentrations are also influenced by obesity, diabetes, diet, and lipid- and glucose-lowering treatment. Medication subclasses were not included separately in our multivariable model because the relevant subgroups were small, treatment categories overlapped, and additional covariates would have increased the risk of overfitting. Thus, the higher triglyceride concentrations observed in MACS represent an association and cannot be attributed directly or exclusively to autonomous cortisol secretion.

Longitudinal studies have reported higher rates of cardiovascular events or mortality in patients with autonomous cortisol secretion [7,8,9,10]. However, these studies have varied with respect to diagnostic thresholds, patient characteristics, follow-up duration, and adjustment for conventional cardiovascular risk factors. Their results therefore should not be interpreted as indicating a uniform risk across all patients with MACS. Because the present study was neither longitudinal nor designed to evaluate cardiovascular events, it cannot establish the prognostic relevance of the hormonal and metabolic differences observed.

Previous echocardiographic studies have identified structural and functional cardiac abnormalities in selected populations with autonomous cortisol secretion. Sbardella et al. observed greater left ventricular mass, a higher prevalence of diastolic dysfunction, and increased arterial stiffness in patients with possible autonomous cortisol secretion compared with patients with NFAI. In that cohort, post-1-mg DST cortisol explained approximately 14% of the variance in left ventricular mass index [17]. Other studies of adrenal incidentalomas have also described markers of subclinical cardiovascular involvement, although their findings have been inconsistent [16].

More recently, MACS was associated with greater left ventricular hypertrophy and poorer diastolic function in patients with aldosterone-producing adenomas [15]. In addition, clinical and experimental data have also suggested that autonomous cortisol secretion may promote vascular calcification in the presence of hyperaldosteronism [23]. These studies, however, were conducted in patients with primary aldosteronism. Because aldosterone and cortisol may exert additive or interacting cardiovascular effects, such findings cannot be directly extrapolated to patients with isolated MACS or NFAI.

In our cohort, no consistent echocardiographic phenotype distinguished MACS from NFAI. Left atrial dimensions and volume, left ventricular internal diameters, LVEF, left ventricular mass and mass index, relative wall thickness, posterior wall thickness, E/A ratio, tissue Doppler e′ velocities, E/e′ ratio, and GLS were comparable between the groups. Although mitral E-wave velocity was lower and interventricular septal thickness differed modestly in the MACS group, these isolated findings were not supported by concordant differences in related structural or diastolic indices and therefore did not form a coherent echocardiographic pattern. Considering the number of echocardiographic comparisons, the modest sample size, and the possibility of residual confounding, these observations should be considered exploratory rather than evidence of subclinical diastolic dysfunction or early cardiac remodeling in MACS.

GLS was likewise comparable between the groups. The mean between-group difference was 0.0 percentage points (95% CI, −1.0 to 1.0; *p* = 0.846). This result indicates that no difference was detected with the precision available in the present sample; it does not establish equivalence. The confidence interval remains compatible with a smaller, potentially clinically relevant difference, and a type II error cannot be excluded. Thus, the data show no statistically significant between-group difference in myocardial deformation rather than proof of identical myocardial function.

The adjusted analysis further supported a cautious interpretation. Diagnostic group was not independently associated with GLS after adjustment for age, sex, BMI, hypertension, diabetes mellitus, and continuous post-1-mg DST cortisol concentration. By contrast, age and diabetes mellitus were independently associated with GLS. These findings highlight the contribution of established demographic and metabolic factors to myocardial deformation and indicate that they should be considered when assessing possible cardiac involvement in adrenal incidentalomas.

Because cortisol autonomy represents a biological continuum, hormonal indices were also evaluated as continuous variables in the entire cohort. Post-1-mg DST cortisol, basal cortisol, ACTH, DHEA-S, and 24 h urinary free cortisol were not significantly associated with GLS. Adrenal lesion size was also unrelated to GLS in the whole cohort and within the MACS and NFAI groups. Accordingly, the present analyses did not identify a dose–response relationship between the biochemical degree of cortisol autonomy or lesion size and myocardial deformation. These findings accord with the absence of a significant between-group difference in GLS. However, the modest sample size and restricted range of hormonal exposure may have limited the ability to detect small continuous associations. Consequently, the cardiovascular effects of mild cortisol excess and the underlying biological mechanisms proposed previously should be regarded as literature-based hypotheses rather than mechanisms demonstrated by our data.

In the unadjusted analyses, nominal associations were identified between GLS and selected clinical or metabolic variables, including an association with triglyceride concentration in the NFAI subgroup. None remained statistically significant after Benjamini–Hochberg false discovery rate correction; therefore, they should be considered strictly exploratory and hypothesis generating. Moreover, nominal significance in one subgroup but not another does not establish a difference between groups. Demonstrating such heterogeneity would require a prespecified interaction analysis with adequate statistical power. As GLS was expressed as a signed negative value, less negative values represent worse myocardial deformation, and the direction of each association should be interpreted accordingly.

Several biological pathways could plausibly link persistent cortisol exposure to cardiometabolic and cardiovascular abnormalities. Signaling through glucocorticoid and mineralocorticoid receptors may affect sodium homeostasis, vascular tone, insulin sensitivity, lipid metabolism, endothelial function, and oxidative stress [11,12,13,14]. Chronic metabolic dysfunction may also promote low-grade inflammation and interfere with the resolution of inflammatory responses, thereby contributing to vascular and myocardial injury [24]. In addition, inflammasome-related pathways have been implicated in fibroblast activation and cardiac fibrosis in cardiometabolic disorders [25]. Although these mechanisms provide a relevant conceptual framework, they were not directly evaluated in the present study and should not be interpreted as explanations established by our findings.

Emerging interventional evidence suggests that some cardiometabolic abnormalities associated with MACS may be modifiable. In the randomized COAR study, selected patients with MACS who underwent adrenalectomy experienced more favorable changes in weight, glucose control, and blood pressure than those managed conservatively [26]. These results complement earlier evidence suggesting potential cardiometabolic benefits of adrenalectomy in appropriately selected patients [20,21]. Nevertheless, improved metabolic control cannot be assumed to translate into improved myocardial deformation. Because we did not evaluate adrenalectomy, serial echocardiographic changes, or cardiovascular outcomes, the present study cannot determine whether treatment of cortisol autonomy modifies GLS or cardiac remodeling.

From a clinical perspective, our results reinforce the importance of comprehensive evaluation and management of hypertension, diabetes, dyslipidemia, obesity, and other conventional cardiovascular risk factors in patients with MACS. However, the absence of an independent association of diagnostic group or continuous cortisol indices with GLS does not support using GLS alone for cardiovascular risk stratification in this population. Decisions concerning follow-up or adrenalectomy should remain individualized and should incorporate the degree and persistence of cortisol autonomy, the presence and severity of potentially cortisol-related comorbidities, age, general health, and patient preferences. Collaboration between endocrinology and cardiology may be valuable for patients with multiple cardiometabolic comorbidities or suspected cardiovascular involvement.

This study has several limitations. First, its retrospective, cross-sectional, single-center design limits generalizability and precludes conclusions regarding causality, temporal progression, and long-term cardiovascular outcomes. Second, the sample size was modest, and no formal a priori sample-size calculation was undertaken. Therefore, the study may have been underpowered to detect small but clinically meaningful differences in myocardial deformation; consequently, the nonsignificant GLS result should not be interpreted as evidence of equivalence. Third, although the 1 mg overnight DST was the principal test used for classification and ACTH independence was confirmed, results of the 2-day low-dose DST were unavailable for some participants because of the retrospective design. Persistence and the full biochemical spectrum of cortisol autonomy therefore could not be evaluated comprehensively in every patient.

Fourth, residual confounding remains possible. Although the multivariable model included major demographic and clinical covariates, individual medication subclasses were not entered separately because treatment categories overlapped, subgroup sizes were small, and further model expansion would have increased the risk of overfitting. Antihypertensive, glucose-lowering, and lipid-lowering treatments may have influenced the metabolic and echocardiographic findings. Twelve patients with nonobstructive coronary atherosclerosis were included in the primary cohort. Although their exclusion in a sensitivity analysis did not materially alter the principal results, residual cardiovascular confounding cannot be ruled out completely.

Fifth, all echocardiographic and strain measurements were performed by a single experienced reader, and formal intraobserver and interobserver reproducibility assessments were not performed. Measurement variability, particularly for speckle-tracking-derived GLS, therefore cannot be excluded. The use of a single ultrasound vendor and analysis platform may also restrict external reproducibility. Furthermore, cardiac magnetic resonance imaging was not performed; thus, myocardial fibrosis and other tissue-level abnormalities not apparent on echocardiography could not be assessed.

Finally, several exploratory correlations were examined. Although false discovery rate correction was applied, none of the nominal associations remained statistically significant, limiting their inferential value. The study also lacked prospective cardiovascular outcome data and direct assessments of inflammation, oxidative stress, endothelial function, and myocardial fibrosis. Therefore, the mechanistic and prognostic implications of the observed hormonal and metabolic differences should be investigated in larger prospective studies incorporating repeated hormonal assessments, standardized cardiovascular imaging, and longitudinal evaluation of clinical outcomes.

In conclusion, patients with MACS had distinct hormonal characteristics and higher triglyceride concentrations than patients with NFAI. However, no statistically significant between-group difference was detected in GLS (NFAI, −21.9 ± 2.2% vs. MACS, −21.9 ± 1.9%; mean difference, 0.0 percentage points; 95% CI, −1.0 to 1.0; *p* = 0.846), and most conventional structural and diastolic echocardiographic parameters were comparable. Continuous measures of cortisol autonomy and adrenal lesion size were not significantly associated with GLS, and nominal exploratory correlations did not remain significant after false discovery rate correction. Thus, endocrine and metabolic differences were observed without a concurrently detectable difference in myocardial deformation. These findings should not be regarded as evidence of cardiac equivalence or as demonstrating early cardiovascular alterations. Larger prospective longitudinal studies incorporating repeated hormonal assessment, standardized cardiovascular imaging, and clinical outcome evaluation are needed to clarify the long-term cardiac implications of MACS.

## Figures and Tables

**Table 1:** Clinical characteristics of the patients.

Parameter	Mean ± SD or Median (IQR)	
**Age (y)**		58.0 ± 8.4
**BMI**		29.4 ± 4.8
**Gender**	Male	23 (34.3)
	Female	44 (65.7)
**BMI**	Normal weight	13 (19.4)
	Overweight	27 (40.3)
	Obese	27 (40.3)
**Diagnosis**	NFAI	32 (47.8)
	MACS	35 (52.2)
**Comorbidity**	No	15 (22.4)
	Yes	52 (77.6)
**HT**	No	32 (47.8)
	Yes	35 (52.2)
**DM**	No	45 (67.2)
	Yes	22 (32.8)
**Hyperlipidemia**	No	59 (88.1)
	Yes	8 (11.9)
**Non-obstructive coronary atherosclerosis**	No	55 (82.1)
	Yes	12 (17.9)
**Osteoporosis**	No	63 (94)
	Yes	4 (6)
**Patients receiving medication**	No	15 (22.4)
	Yes	52 (77.6)

Results are presented as mean ± standard deviation or median (IQR) or *n* (%). BMI: body, mass index, HT: Hypertension, DM: Diabetes mellitus, NFAI: nonfunctioning adrenal incidentaloma, MACS: mild autonomous cortisol secretion.

**Table 2:** Laboratory parameters of the patients.

Parameter	Mean ± SD or Median (IQR)
**Glucose (mg/dL)**	96 (87–114)
**HOMA-IR**	2 (1.3–3.3)
**HbA1C (%)**	5.8 (5.5–6.4)
**GFR (mL/min/1.73 m^2^)**	99 (92–106)
**Creatinine (mg/dL)**	0.7 (0.6–0.8)
**ALT (U/L)**	17 (14–23)
**LDL (mg/dL)**	125.5 ± 42.1
**TG (mg/dL)**	129 (91–184)
**TSH (µIU/mL)**	1.8 (1.1–2.7)
**Na (mmol/L)**	139.4 ± 2.4
**K (mmol/L)**	4.4 ± 0.4
**Cortisol (µg/dL)**	12.0 ± 4.3
**ACTH (pg/mL)**	15.6 ± 8.8
**Post-1-mg DST cortisol (µg/dL)**	2.4 ± 1.4
**Aldosterone (ng/dL)**	13.0 ± 8.5
**Renin activity (ng/mL/h)**	1.2 (0.7–2.6)
**24 h urinary free cortisol (µg/24 h)**	195.9 ± 78.3
**DHEA-S (µg/dL)**	63.5 ± 43.8

Results are presented as mean ± standard deviation or median (IQR) or *n* (%). DST: Dexamethasone Suppression Test.

**Table 3:** Comparison of clinical, echocardiographic and laboratory parameters between patients with MACS and patients with NFAI.

Parameter	NFAI	MACS	*p*
	Mean ± SD or Median (IQR)	Mean ± SD or Median (IQR)	
**Age**	57.4 ± 9.1	58.6 ± 7.8	0.575
**BMI**	28.3 (26.1–30.6)	29.5 (27.3–33.8)	0.404 ^+^
**Glucose (mg/dL)**	98 (89.5–113)	95 (86–118)	0.514 ^+^
**HOMA-IR**	2 (1.2–2.9)	2.5 (1.4–3.7)	0.158 ^+^
**HbA1C (%)**	5.9 (5.5–6.4)	5.8 (5.5–6.4)	0.678 ^+^
**GFR (mL/min/1.73 m^2^)**	97.5 (92–105.5)	100 (93–106)	0.683 ^+^
**Creatinine (mg/dL)**	0.7 (0.6–0.8)	0.7 (0.6–0.8)	0.498 ^+^
**ALT (U/L)**	19 (14–28)	17 (14–19)	0.148 ^+^
**LDL (mg/dL)**	117 ± 34.5	133.2 ± 47.2	0.116
**TG (mg/dL)**	102 (78.5–148.5)	154 (106–222)	**0.004** ^+^
**TSH (mIU/L)**	1.6 (1–2.7)	1.8 (1.1–2.9)	0.478 ^+^
**Na (mmol/L)**	139.1 ± 2.4	139.8 ± 2.3	0.248
**K (mmol/L)**	4.5 ± 0.3	4.3 ± 0.4	0.084
**Cortisol (µg/dL)**	12.4 ± 5.1	11.6 ± 3.5	0.445
**ACTH (pg/mL)**	18.6 ± 9.7	13.0 ± 7.1	**0.008**
**Post-1-mg DST cortisol (µg/dL)**	1.2 ± 0.5	3.4 ± 1.3	**<0.001**
**Aldosterone (ng/dL)**	13.5 ± 9.6	12.6 ± 7.5	0.678
**Renin activity (ng/mL/h)**	1.3 (0.8–2.5)	1 (0.7–2.6)	0.665 ^+^
**24 h urinary free cortisol (µg/24 h)**	204.2 ± 79.2	188.2 ± 77.8	0.413
**DHEA-S (µg/dL)**	82.8 ± 48	46.4 ± 31.6	**<0.001**
**Size (mm)**	23.6 ± 7.9	29.4 ± 11.4	**0.019**
**GLS, %**	−21.9 ± 2.2	−21.9 ± 1.9	0.846

Results are presented as mean ± standard deviation or median (IQR) or *n* (%). NFAI: nonfunctioning adrenal incidentalomas, MACS: mild autonomous cortisol secretion, GLS: global longitudinal strain. ^+^ Mann–Whitney U test was performed, otherwise Student’s *t* test was used. GLS values are reported as signed percentages; more negative values indicate better systolic deformation. *p*-value < 0.05 represented statistically significancy.

**Table 4:** Comparison of echocardiographic parameters between NFAI and MACS patients.

Echocardiographic Parameter	NFAI	MACS	*p*
	Mean ± SD or Median (IQR)	Mean ± SD or Median (IQR)	
**LA diameter (mm)**	38 (36–40)	38 (37–41)	0.498
**LA area (cm^2^)**	22 (21–23)	22 (21–23)	0.486
**LA volume (mL)**	44 (40.5–46.5)	42 (40–46)	0.359
**LAVI (mL/m^2^)**	22.9 ± 2.3	23.1 ± 3	0.817
**IVS (mm)**	10 (9–10)	10 (9–11)	**0.040**
**PW (mm)**	9 (9–10)	10 (9–10)	0.271
**LVEDD (mm)**	47 (45–48)	46 (44–48)	0.390
**LVESD (mm)**	29 (27.5–30)	29 (27–30)	0.571
**LVEF (%)**	68 (66.5–72)	68 (68–70)	0.819
**RWT**	0.39 (0.38–0.43)	0.42 (0.38–0.45)	0.283
**LV mass (g)**	169.9 ± 19.6	163 ± 24.2	0.203
**LVMI (g/m^2^)**	88.7 ± 8.2	87.8 ± 10.1	0.679
**Mitral E (cm/s)**	63.3 ± 12.3	56.9 ± 9.2	**0.019**
**Mitral A (cm/s)**	66 (54–72.5)	65 (52–74)	0.900
**E/A**	0.9 (0.7–1.4)	0.9 (0.7–1.1)	0.275
**Mitral lateral e′ velocity (cm/s)**	11 (9–13)	10 (9–12)	0.200
**Mitral septal e′ velocity (cm/s)**	11 (9.5–12)	10 (9–11)	0.411
**E/e′**	5.8 (5.4–6.4)	5.4 (4.8–6)	0.120

**Table 5:** Relationship between GLS and laboratory parameters in patients with NFAI, MACS, and total.

	NFAI	MACS	Total Patients
**Glucose (mg/dL)**	−0.20 (0.323)	0.24 (0.232)	0.25 (0.040)
**HOMA-IR**	0.10 (0.663)	0.19 (0.431)	0.03 (0.858)
**HbA1C (%)**	−0.12 (0.577)	0.30 (0.140)	0.25 (0.042)
**GFR (mL/min/1.73 m^2^)**	−0.33 (0.100)	0.08 (0.684)	−0.36 (0.003)
**Creatinine (mg/dL)**	0.30 (0.136)	−0.09 (0.669)	0.05 (0.665)
**ALT (U/L)**	0.00 (0.994)	0.07 (0.731)	−0.18 (0.135)
**LDL (mg/dL)**	−0.08 (0.710)	0.06 (0.764)	−0.25 (0.046)
**TG (mg/dL)**	−0.43 (0.028)	0.02 (0.937)	−0.24 (0.050)
**TSH (mIU/L)**	−0.22 (0.289)	0.17 (0.418)	−0.02 (0.868)
**Na (mmol/L)**	0.21 (0.295)	0.22 (0.271)	−0.06 (0.659)
**K (mmol/L)**	0.14 (0.507)	−0.02 (0.941)	0.19 (0.124)
**Cortisol (µg/dL)**	0.17 (0.407)	−0.32 (0.114)	0.09 (0.493)
**ACTH (pg/mL)**	0.24 (0.229)	0.02 (0.937)	0.06 (0.657)
**Post-1-mg DST cortisol (µg/dL)**	−0.10 (0.618)	0.06 (0.777)	0.04 (0.752)
**Aldosterone (ng/dL)**	0.28 (0.190)	0.23 (0.291)	−0.03 (0.844)
**Renin activity (ng/mL/h)**	0.33 (0.122)	0.12 (0.587)	−0.07 (0.614)
**24 h urinary free cortisol (µg/24 h)**	−0.19 (0.360)	0.03 (0.893)	0.01 (0.918)
**DHEA-S (µg/dL)**	0.20 (0.331)	−0.01 (0.947)	−0.09 (0.471)
**Size (mm)**	−0.09 (0.660)	0.01 (0.979)	0.002 (0.990)

NFAI: nonfunctioning adrenal incidentalomas, MACS: mild autonomous cortisol secretion. Results are presented as correlation coefficients and *p*-values r (p).

**Table 6:** Multivariable linear regression analysis for predictors of GLS.

Variable	Unstandardized *β*	95% CI	Standardized *B*	*p*	VIF
Groups (NFAI/MACS)	0.445	−0.762 to 1.653	0.110	0.463	2.385
Age (y)	0.152	0.099 to 0.205	0.630	**<0.001**	1.293
BMI (kg/m^2^)	0.009	−0.105 to 0.088	−0.020	0.860	1.394
Sex	0.568	−0.382 to 1.519	0.134	0.236	1.346
Post-1-mg DST Cortisol (µg/dL)	−0.411	−0.831 to 0.009	−0.292	0.055	2.395
Hypertension	0.367	−0.551 to 1.285	0.091	0.427	1.378
Diabetes Mellitus	1.232	0.361 to 2.103	0.287	**0.006**	1.107

Diagnostic group was coded as NFAI = 0 and MACS = 1. *p*-value < 0.05 represented statistically significancy.

## Data Availability

The data presented in this study are available on request from the corresponding author due to privacy and ethical restrictions. The data are not publicly available to protect patient confidentiality.
